# Recent Advances in Tick Antigen Discovery and Anti-Tick Vaccine Development

**DOI:** 10.3390/ijms24054969

**Published:** 2023-03-04

**Authors:** Muhammad Nadeem Abbas, Mohamed Amine Jmel, Imen Mekki, Ingrid Dijkgraaf, Michail Kotsyfakis

**Affiliations:** 1 State Key Laboratory of Silkworm Genome Biology, Southwest University, Chongqing 400716, China; 2 Laboratory of Genomics and Proteomics of Disease Vectors, Institute of Parasitology, Biology Centre, Czech Academy of Sciences, 37005 Ceske Budejovice, Czech Republic; 3 Department of Biochemistry, CARIM, Maastricht University, 6229 ER Maastricht, The Netherlands

**Keywords:** vaccinomics, antigen candidates, anti-tick vaccine, tick control

## Abstract

Ticks can seriously affect human and animal health around the globe, causing significant economic losses each year. Chemical acaricides are widely used to control ticks, which negatively impact the environment and result in the emergence of acaricide-resistant tick populations. A vaccine is considered as one of the best alternative approaches to control ticks and tick-borne diseases, as it is less expensive and more effective than chemical controls. Many antigen-based vaccines have been developed as a result of current advances in transcriptomics, genomics, and proteomic techniques. A few of these (e.g., Gavac^®^ and TickGARD^®^) are commercially available and are commonly used in different countries. Furthermore, a significant number of novel antigens are being investigated with the perspective of developing new anti-tick vaccines. However, more research is required to develop new and more efficient antigen-based vaccines, including on assessing the efficiency of various epitopes against different tick species to confirm their cross-reactivity and their high immunogenicity. In this review, we discuss the recent advancements in the development of antigen-based vaccines (traditional and RNA-based) and provide a brief overview of recent discoveries of novel antigens, along with their sources, characteristics, and the methods used to test their efficiency.

## 1. Introduction

Ticks are ectoparasites that infest humans and animals and are responsible for significant economic losses. They are the second most important vectors for the transmission of diseases in humans after mosquitoes [1,2]. They are also one of the most important vectors for the transmission of diseases that impact the global cattle industry and pets [3,4,5]. Ticks have few natural enemies, making it challenging to control tick infections. Chemical acaricides have been only partially effective, with a number of nontarget disadvantages, including the selection of acaricide-resistant ticks and contamination of the environment and animal products with chemical residues [6]. In addition, to control tick-borne diseases, some antigen-based vaccines are used in various countries; however, new and more effective approaches are needed, including the development of new vaccines that target tick infestations and pathogen infections [7,8]. 

Traditionally, the “isolate–inactivate–inject” principle has played a crucial role in designing and developing a vaccine for the control of parasites/pathogens. First-generation vaccines were composed of pathogens that were alive, attenuated, or killed. Second-generation vaccines consisted of purified parasite/pathogen components and were developed as a result of advances in cell culture, polysaccharide chemistry, recombinant DNA technology, and immunology [9,10]. The advancement of genomics and other “omics” over the last two decades has resulted in the development of a “third generation” of vaccines, based on technologies such as functional omics, reverse vaccinology, and the systems biology approach. In order to overcome the limitations of the conventional vaccine development approaches, vaccine development has become more tailored, with a focus on the antigen moieties that are targeted by the protective immune responses [11,12], with the broad perspective of the pathogen and its interaction with the host immune system [13]. Hence, modern vaccinology relies increasingly on novel omics approaches utilizing high-throughput cutting-edge technologies, such as genomics, transcriptomics, and proteomics, along with advances in basic immunology, host–pathogen biology, immunomics, advanced bioinformatics, and computational modelling, and improved understanding and technological innovations.

Compared to using chemicals, vaccination is a wise option because it is environmentally safe and cost-effective to control tick infestation [12,14]. Although vaccination is a rational strategy for controlling tick infestation, only a few vaccines have been commercialized so far, with minimal concern given to the induction of cross-reactive immunity against tick species [15]. To develop new vaccines, it is crucial to identify and characterize novel antigen candidates that would be more conserved and have the ability to induce cross-reactive immunity in the host species. The goal of this review is to provide an overview of traditional and RNA-based vaccines and the possibility of their application and novel antigens that have the potential to be exploited as promising antigen candidates for vaccine development.

## 2. Identification of Antigens: A Road Map to Develop an Anti-Tick Vaccine 

The identification of antigens is paramount for the development of an anti-tick vaccine. It is crucial to understand the molecular mechanisms associated with the host–parasite–pathogen interactions to identify antigen candidates that are likely to serve as candidates/targets for the development of a vaccine. The ideal antigen candidate is one that induces long-lasting and effective immune responses in the host [16,17]. Many studies have been carried out since Allen and Humphreys published their findings in 1979, employing a range of antigens, including whole tick homogenates and internal organs, to induce varying levels of immunity against ticks [16]. 

**Figure 1:**
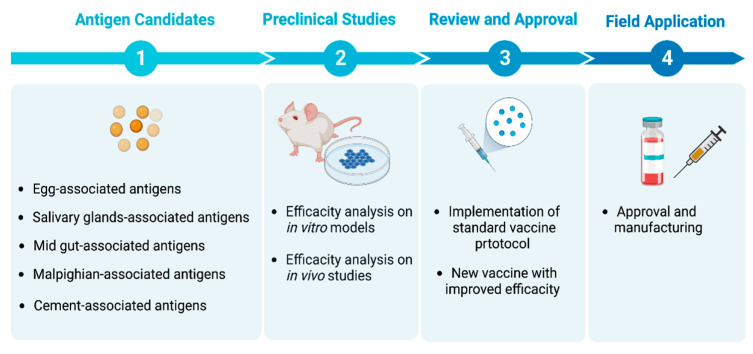
An overview of the distribution and efficacy evaluating scheme of tick vaccine antigens targets for the prevention of tick infestations and tick-borne diseases.

Several new possibilities have emerged for predicting, screening, and identifying antigens protective against tick infestations since *Ixodes scapularis*, the first tick species to be sequenced [18]. There are now many nucleotide and protein databases available from different tick tissues and developmental stages, and a wide variety of stimuli that affect ticks, such as tick feeding or infection with pathogens [17,19], are known. The probability of selecting protective antigen candidates derived from ticks for the control of tick infestation and pathogen infection has also increased as a result of recent advances in omics technologies (i.e., transcriptomics, proteomics, and metabolomics) [20]. In addition, the use of reverse vaccinology (RV), or vaccinomics, has allowed the discovery of new vaccine antigen candidates [20]. As a result of this, synthetic and recombinant proteins have been evaluated and demonstrated to be able to induce some level of protective immunity. The purpose of this section is to discuss antigen candidates originating from different tissues which have been identified, assessed for their efficacy, and are being considered as potential candidates for the development of an anti-tick vaccine, based on the available literature (Table 1 and Figure 1).

### 2.1. Egg-Associated Antigen Candidates 

Egg yolk is an essential component for the development of ticks, since it serves as a reservoir of various proteins that play a crucial role during the embryonic development of these arthropods [21,22]. As in insects, yolk proteins are synthesized in the fat body of ticks [21,23]. The degradation of the yolk is carried out by various types of enzymes which are found in eggs. Boophilus Yolk pro-Cathepsin (BYC) is an example of a yolk proteinase that has been isolated from *R. microplus* eggs, and has been reported to be involved in the embryogenesis process of the tick. In particular, these enzymes play a key role in the degradation of vitelline, a major proteinaceous component of egg yolk [21]. BYC was first isolated by da Silva Vaz Jr et al. [24] from *R. microplus* eggs, and was then inoculated into cattle to determine its role in the induction of host immunity. This enzyme was found to provide partial protection against ticks and trigger a protective immune response in cattle, but its efficacy was between 14% and 36%. A subsequent study expressed recombinant BYC protein in a prokaryotic expression system (*E. coli*). Interestingly, the recombinant protein showed an overall higher efficacy (25.24%) compared to the enzyme directly isolated from egg yolk [25,26]. It appeared that various factors may affect the efficacy of this protein, for example, the method of preparation of BYC protein can influence the protein structure and ultimately its functions. Furthermore, this variation may be also associated with the tick strain or other experimental conditions [24]. 

Vitellin, a lipoglycoprotein also occuring in the egg yolk similar to other yolk proteins, is synthesized in the fat bodies of arthropods [27,28]. In ticks, vitellin or vitellogenins have been shown to be crucial for egg development and oviposition as demonstrated by the silencing of three vitellogenin genes in *H. longicornis* [29]. Vitellin protein was purified from tick eggs as a non-covalent complex of six polypeptides of high molecular weight (44–107 kDa). Parallel to this study, an 80 kDa glycoprotein (GP80) was isolated and purified from *R. microplus* larvae. Both proteins were then inoculated to investigate their efficacy. Vitellin and GP80 vaccination showed an overall 68% efficacy, suggesting that a vaccine containing both antigens can induce an immune response and also provide partial protection against *R. microplus* in sheep hosts [28]. Remarkably, when recombinant hexahis-GP80 (HH-GP80), which was incorrectly folded and not glycosylated, was injected into the host under the same experimental conditions, it displayed no efficacy [28]. Based on the findings of the above study, it appears that vaccination of vitellin and GP80 can elicit immune responses in sheep and may partially protect sheep against the tick *B. microplus*. The correct folding of HH-GP80 is crucial for its activity, since protective epitopes are associated with the folding of the protein and/or the oligosaccharides attached to it, and these epitopes are essential for its activity.

Vitellin degrading cysteine endopeptidase (VTDCE) is another egg-associated enzyme which was identified and isolated by Seixas et al. [30]. Similar to BYC, this enzymatic protein is not synthesized in the ovary of *R. microplus* and is implicated in vitellin hydrolysis, thereby providing nutrients to developing embryos. However, both enzymes were found to regulate vitellin hydrolysis differently [30]. The same research group later analyzed purified VTDCE protein as an antigen and found that this protein also provides partial protection against ticks, as the immunization of livestock resulted in 21% efficacy and a 17.6% reduction in the weight of fertile eggs [31]. The egg-associated proteins BYC and VTDCE provided limited protection to the host against tick infestation, and therefore seem to be not suitable antigen candidates when used alone in a vaccine.

### 2.2. Salivary Gland-Associated Antigen Candidates 

Ticks contain an angiotensin-converting enzyme-like protein that can control blood pressure by regulating fluid volume, similar to the angiotensin-converting enzyme in mammals [32,33]. This control allows the tick to feed continuously on the host’s blood. The salivary glands and midgut of tick *B. microplus* contain a low abundance glycoprotein, which is named Bm91 [32]. Bm91 is currently not included in commercial anti-tick vaccines, but it is considered to be a candidate for controlling ticks [34]. When the recombinant Bm91 protein was assessed alone under field conditions with natural tick infestation, the results were disappointing, as this protein showed only 6% efficacy, which seems to inappropriate for the development of a vaccine for tick control [35]. However, when recombinant Bm91 protein which was produced in *E. coli* was combined with Bm86 (an antigen candidate that is used in commercial vaccines) and then this protein combination was used as a vaccine, the results were much more promising, since the Bm91 addition enhanced the efficacy of the Bm86 antigen [33], suggesting that the combination of these two proteins (Bm91 and Bm86) seems to be an effective strategy to develop a new anti-tick vaccine.

Transcriptomic and differential gene expression analyses of salivary glands have shown that the genome of tick (e.g., *R. microplus* and *Dermacentor andersoni*) species comprises a protein sequence named flagelliform silk protein [36,37]. The characterization of differential gene expression in the salivary glands of *R. microplus* in response to *A. marginale* infection highlighted the molecular mechanisms of how the tick interacts with the pathogen. Subsequent functional studies have shown that flagelliform silk protein (SILK) may play a crucial role in the infection and multiplication of *A. marginale* in ticks. An interaction between tick- and pathogen-derived molecules is involved in the multiplication of *A. marginale* in salivary gland cells [36,38]. Following this study, it was proposed that flagelliform silk protein could be a suitable antigen candidate to develop a vaccine. For this purpose, Merino et al. [14] produced recombinant flagelliform silk protein and analyzed its antigenic activity by injecting it into a cattle host. The recombinant protein was found to be an excellent antigenic candidate, as it provided 62% protection against tick infestation and tick-borne infection (e.g., babesiosis) in cattle. Vaccination with flagelliform silk protein reduced the multiplication of *A. marginale* in cattle. Theantigen-specific antibody titers correlated with reduced tick infestations and pathogen infection, indicating that the effect of the vaccine is a result of the antibody response. Furthermore, the expression of gene-encoding vaccine antigens in ticks feeding on cattle was also affected by vaccination and co-infection with *A. marginale* and *B. bigemina*. Thus, it appears that vaccines using tick proteins that are involved in vector–pathogen interactions can be effective in both controlling tick infestation and preventing pathogen infection at the same time [14].

Salp15 is an immune suppressive salivary protein of *I. scapularis* with a molecular weight of 15 kDa that inhibits the activation of CD4+ T cells, the complement activity, cytokine production, and the dendritic cell function in the host [39,40,41]. Subsequent studies investigated the molecular mechanism of Salp15. The outer surface protein, OspC, is produced by *B. burgdorferi* on the outer surface of the cell. The production of spirochetes (*B. burgdorferi* spirochetes) in the midgut of infected ticks is initiated when it feeds on blood from the host, which is then transported to the host. During the exit from the salivary glands and transmission of the *B. burgdorferi* spirochetes to the host, Salp15 physically interacts with OspC on the surface of *B. burgdorferi* spirochetes, which facilitates the survival of spirochetes, pathogen transmission, and host infection [38,42]. Salp15–OspC interaction may thus potentially obscure OspC from the host immune response so that the spirochete is protected from the immune response [38]. Recently, the *Escherichia coli* expression system was used to synthesize Salp15 recombinant protein, and the system was found to be efficient in producing this protein in a considerable yield with good solubility. These characteristics of Salp15 recombinant protein indicate that this has practical application and can be used to generate anti-tick vaccines [41,43,44].

Metalloproteases (MPs) are multifunctional proteins that participate in a wide variety of complex physiological and pathologic processes in living organisms [45]. A number of MPs have been identified in different tick species and are considered to be crucial for the maintenance of blood meal-associated functions in ticks [46,47,48,49]. For example, the salivary glands of ixodid ticks contain MPs that are recognized as key bioactive components in vital physiological functions and are therefore considered for use as potential targets in control strategies to combat these ectoparasites [49]. In order to evaluate the antigenic potential of MPs, Ali et al. (2015) [50] amplified a fragment of the sequence encoding a *R. microplus* MP, expressed it as a recombinant protein, and used the purified form of this protein as a vaccine antigen against *R. microplus* in cattle [50]. The recombinant *R. microplus* MP protein demonstrated an overall efficacy of 60%. In addition, it reduced the number of feeding ticks, the number of eggs produced, and the number of eggs hatched, making [41] it an ideal candidate for anti-tick vaccine development [50]. To further explore suitable antigen candidates from *R. microplus,* Maruyama et al. [51] performed an RNA-seq study on salivary glands at all feeding stages of *R. microplus*, and they detected a fragment from the transcriptome which was similar to MP (Rm239) along with three other genes, including Rm39, Rm76, and Rm180. Application of these proteins as vaccines showed that all of them can inhibit hemostatic responses, suppress the host’s antibody responses, and reduce the tick’s ability to bind to the host by means of a glycine-rich cement protein. Therefore, the authors developed a multicomponent anti-tick vaccine using these four different types of proteins [51]. The immunization of cattle with this multicomponent vaccine resulted in a reduction in the infestation of *R. microplus* by 73.2%, indicating that the formulation of a multi-antigen anti-tick vaccine may be more effective than monocomponent vaccines [51].

Ribosomes, also called protein factories, are components of all living organisms. It has been shown that the ribosomal protein P0 plays a pivotal role in regulating the translational activity of ribosomes and assisting an organism to adjust its metabolism to various environmental conditions. It belongs to a group of acidic proteins that form a stalk-like structure in the largest ribosome subunit of the ribosome [52]. There is evidence that shows that tick saliva contains ribosomal proteins that play a role in evading the defensive mechanisms of the host [53,54,55]. It was recently reported that rabbits vaccinated with recombinant ribosomal protein P0 exhibited strong humoral responses that primarily reduced nymph molting and female reproduction. The protein demonstrated a 57.5% protection against infestations of *O. erraticus*, but did not provide cross-protection against infestations of the African tick *Ornithodoros moubata* [56]. In another study, researchers chemically synthesized a peptide of 20 amino acids, which was derived from the ribosomal P0 protein of *Rhipicephalus* ticks, and successfully conjugated it to the Keyhole Limpet Hemocyanin (KLH) protein of *Megathura crenulate* to serve as an antigen against *R. microplus*, showing 96% efficacy in cattle [57]. In this study, the results suggested that P0 conjugated to KLH is an excellent vaccine. However, the production of such a vaccine will be expensive and may therefore not be cost-effective for livestock. It is therefore essential to conduct further research on the recombinant production of an antigenic vaccine to evaluate its effectiveness and to make its production more economically viable.

Serine protease inhibitors: Attempts to isolate antigens from tick species have identified some serine proteinase inhibitors (serpins), which appeared to have antigen abilities. Serpins are involved in various physiological activities in animals, in particular in cattle, where they influence blood clotting, altering prothrombin time and partially activating thromboplastin time [58,59,60]. Serpins interfere with the immune system of ticks and thereby facilitate the initial feeding process of these parasites [61]. Andreotti et al. [62] isolated and identified *R. microplus* trypsin inhibitors (BmTIs) from larval extracts. To evaluate its antigenic activity, crossbred cattle were vaccinated with BmTI, which was found to interfere with leukocyte migration at the site of larvae fixation [63,64]. Vaccination of calves with BmTI antigens remarkably reduced engorged female tick numbers and their weight, resulting in a 72.8% efficacy against *R. microplus*. This data suggested that BmTI immunization may act in the early phase of larval development [65]. To investigate whether truncated BmTI can also induce immunization, the N-terminal fragment of BmTI was synthesized and showed a lower efficacy (18.4%) in cattle compared to the full-length protein. Thus, immunization with the N-terminal domain is apparently not sufficient to improve the effect of BmTIs on host–parasite interactions [64]. Similarly, when the recombinant *R. microplus* larvae trypsin inhibitors (rRmLTIs) were employed as a vaccine trial, the efficacy (32%) was again low, suggesting that both the truncated or whole recombinant protein are less effective, probably due to a lack of precise folding of the protein in vitro. Overall, these results indicate that trypsin inhibitors seem suitable candidates to produce an effective vaccine; however, the method to produce them on a large scale needs to be improved to enhance its efficacy [66]. Various other serpins have been evaluated as possible anti-tick vaccine candidates from different tick species, including *Amblyomma americanum* (AAS19), *Haemaphysalis longicornis* (HLS2), *Rhipicephalus* (Boophilus) *microplus*, and so on. All of these serpins demonstrated a partial protection to the host; however, the level of protection may vary with tick species and the type of serpin [67,68,69]. 

Combining proteins from one or more ticks into a single polypeptide chain represents an attractive anti-tick vaccination strategy. Therefore, trypsin inhibitors/or serpins combined with immunogenic fragments of other tick proteins can be used as multi-antigen constructs. For example, a chimeric protein containing the recombinant Bm86-Campo Grande antigen (BmCG), rRmLTI, and the heat-labile enterotoxin B subunit from *Escherichia coli* (LTB) as a molecular adjuvant was synthesized. This chimeric RmLTI–BmCG–LTB antigen had a 55.6% efficacy against *R. microplus* in cattle. 

### 2.3. Midgut-Associated Antigen Candidates

Ferritin proteins are important for the physiological storage of iron in a nontoxic but biologically available form. They are crucial for the metabolism of iron from ingested blood during tick feeding [70,71]. So far, two ferritin molecules (Ferritin 1 and Ferritin 2) have been identified and characterized. Ferritin 1 (FER1) is located within cells, where it is involved in the physiological storage of iron. For Ferritin 2 (FER2), there are no functional orthologs in vertebrates. It is mainly expressed in the gut and plays a crucial biological role in iron transport to the salivary glands and ovaries [71]. The FER2 protein has been reported in various tick species including *D. variabilis*, *R. microplus*, *I. ricinus, Haemaphysalis longicornis*, and *I. scapularis* [71,72]. Based on loss-of-functions studies of FER2, it is a promising vaccine candidate, because suppression of this gene impairs tick feeding ability, lowers oviposition, and reduces larval hatching [71]. Besides FER2, FER1 has been shown as a suitable antigen candidate to control a variety of tick species. Hajdusek et al. tested the recombinant FER2 protein of *R. microplus* (RmFER2) to immunize cattle and found that the FER2-based vaccine showed an overall efficacy of 64% [73]. Similarly, the recombinant proteins FER1 and FER2 of *H. longicornis* have been used to immunize rabbits. Both proteins are highly immunogenic and induced host antibody production. Immunizing the host significantly reduced the engorged weight of the infested ticks and reduced the number of eggs and the number of ticks with completely hatched eggs. However, recombinant FER2 caused a greater reduction with a higher efficacy (49%) than recombinant FER1 (34%) [72]. More recently, Manjunathachar and co-workers [74] reported that a calf vaccinated with *H. anatolicum* FER2, a vector of Crimean–Congo hemorrhagic fever, was strongly protected from larval (51.7%) and adult (51.2%) tick infestations, as well as against ticks with FER2 knocked down by RNAi. Many other recent studies have also confirmed that FER2 provides significant protection to the host against tick infestation by using a recombinant protein of FER2 [75,76,77]. The molecular mechanism of protection involves mainly the production of anti-FER2 antibodies in the host body, which are transferred into tick species during the feeding process, and the anti-FER2 antibodies bind to FER2 inside the tick gut cells or hemolymph, thereby preventing FER2 assembly and/or function. It has been recently discovered that predicted antigenic regions on the FER2 protein are conserved across different tick species. This protein can therefore be used to produce a vaccine for cross-species protection [77]. In a recent study, FER2 orthologues in *O. moubata* (OMFER2) and *O. erraticus* (OEFer2) were characterized, and the researchers found that they have high sequence similarity (85.3%). The recombinant form of *O. moubata* Fer2 (tOMFER2) has the ability to elicit strong humoral responses in rabbits. However, in *O. erraticus*, this protein does not exhibit any protective effect, despite the high sequence similarity, which suggests that a slight difference in their sequences may determine whether or not they have a protective effect. In spite of this, the results of this study confirm that OMFER2 has the potential to serve as an antigen candidate for vaccines [78]. 

TROSPA is a tick receptor that is required for spirochete colonization in *I. scapularis*. The *B. burgdorferi* outer surface protein A (OspA) is abundantly produced on these spirochetes and is critical for adhesion to the vector via specific binding to TROSPA [79,80]. In different tick species, including *R. microplus*, *I. scapularis*, and *R. annulatus*, TROSPA may play a role in infection mechanisms and the multiplication of *Babesia* pathogens. In addition, the outer surface proteins OspA and OspB are expressed when the spirochetes enter and reside in ticks [81]. However, their expression is suppressed during transmission to the host, whereas the expressions of OspC and bba52 are upregulated. BBA52, along with the OspC protein of borrelial, has complementary but non-essential roles in the transmission process, as these antigens are all localized in the outer membrane and co-expressed in feeding ticks [82,83,84]. The biological function of the receptor is unknown, but binding of OspA to TROSPA is necessary in ticks for the bacterium *B. burgdorferi* to colonize the tick gut, which supports the bacterial infection in the vector [79]. Infection of *B. burgdorferi* induces the production of particular tick genes (TROSPA and salp15), which can be targeted to inhibit the transmission of *Borrelia spirochetes* and other tick-borne microbes [80,85]. Blocking TROSPA with TROSPA antisera or via RNAi reduces the adherence of *B. burgdorferi* to the gut of *I. scapularis*, and thus reduces the bacterial colonization of the vector and potentially pathogen transmission to the host [79]. As a result of this interaction, recombinant TROSPA was analyzed in cattle as an antigen vaccine to control tick infestation and pathogen transmission, but it did not affect tick feeding or fecundity [14].

Aquaporins (AQPs) or transmembrane water channels play a major role in water homeostasis and cryoprotection [86,87]. They are evolutionarily highly conserved members of a larger family of major intrinsic proteins. They form pores in the cell membrane that transport water or other solutes [86,88,89]. In addition to transporting water and small neutral solutes, AQPs are involved in numerous physiological processes [90]. In ticks, AQPs have been reported in the digestive track, Malpighian tubules, and also in salivary glands [91]. AQPs reduce the host blood volume in tick guts, an important physiological function since ticks ingest large volumes of blood relative to their size and weight [62]. A fragment of an aquaporin from *R. microplus* engorged females has been isolated and subsequently recombinantly produced and designated as an RmAQP1 vaccine [62]. This vaccine was tested in two cattle pen trials for efficacy against *R. microplus,* demonstrating 68% and 75% efficacy. This suggests that RmAQP1 may be a potential vaccine antigen [62] and that aquaporins can be used in anti-tick vaccines [62]. In a recent study on the RmAQP2 of the same species, it has been demonstrated that cattle vaccinated with the synthetic peptide of the extracellular domains of the RmAQP2 were able to reduce the number of ticks feeding to repletion by 25% overall, suggesting that this target (RmAQP2) may be a useful component of a vaccine cocktail against tick bites [92]. Another study on *I. ricinus* confirmed the efficacy of the tick AQP antigens for the control of tick infestations by showing the effect of IrAQP and CoAQP vaccination on *I. ricinus* tick larvae in rabbits. The efficacy of the vaccine containing the AQP conserved region present in the CoAQP antigen was higher than that of the IrAQP vaccine [93]. Furthermore, vaccination with synthetic immunogenic peptides derived from *Ornithodoros erraticus* AQPs (OeAQP and OeAQP1) provided significant protection to cattle against the homologous species *O. erraticus*, but the cross-species protection against *Ornithodoros moubata* was lower [94]. Besides, some other studies have also identified AQPs from different species, including *O. moubata* and Ixodid ticks, with bioinformatics analyses suggesting that these AQPs have a good potential to be used as a vaccine. Therefore, further experimental evidence is required to confirm the antigen potential of these AQPs [95,96].

*I. ricinus* is one of the tick species responsible for the growing prevalence of tick-borne diseases in companion animals in Europe [4]. The effect of the AQP-based vaccines on *I. ricinus* larvae infestation and molting could result in a reduction in tick infestations in vaccinated animals and supports that CoAQP might be a candidate protective antigen for the control of different tick species feeding on the same host.

A study using expression library immunization against a mouse model of tick infestations showed that the 4D8 protein, later named subolesin (SUB), is a potential antigen that could be used as a vaccine against *I. scapularis* [97]. It was found that the sequences of the gene and protein of SUB are conserved across invertebrates and vertebrates. In addition, this gene has been identified and characterized in different tick species, and was found to be expressed at different developmental stages and in different tissues of adult ticks [98]. Due to SUB’s broad distribution, it was proposed to be a good antigen vaccine candidate. The antigen potential of SUB has previously been investigated in cattle using recombinant proteins, and it was found that SUB can protect (51% efficacy) against ticks. Furthermore, a combination of SUB vaccination and tick autocidal control following SUB gene knockdown in ticks feeding on cattle to control *R. microplus*, attained 75% efficacy after treatment [99,100]. Furthermore, Shakya and co-workers produced recombinant SUB of *R. microplus* and used this recombinant protein to immunize bovine. These ruminants were then challenged with *R. microplus* larvae. In addition, the efficacy of this protein against another geographically different tick strain was assessed. The efficacy of recombinant SUB ranged from 32.7% to 44.1% and indicated a high sequence homology between tick strains from Mexico and India [101]. In another study, recombinant SUB was synthesized as a chimeric protein with MSP1a and subsequently applied to cattle to control *R. microplus.* Surprisingly, this chimeric protein demonstrated an 81% efficacy [102]. As a result of the successful and promising results of SUB application, the combination of this antigen with Bm86 was tested and assumed to give better results, but the overall efficacy did not support the use of this combination as a vaccine. Although it has been shown that high levels of specific antibodies are activated for each antigen when two antigens are administered simultaneously, they are separated into different formulations and used at different inoculation sites in the animal [103,104].

Formerly known as ligandins, glutathione S-transferases (GSTs) form a family of multifunctional proteins widely distributed in the animal kingdom. These enzymatic proteins play a role in intracellular transport, digestion, production of prostaglandins, detoxification of both endogenous and exogenous substances, and defense against oxidative stress. GST expression levels are increased in organisms when exposed to insecticides and acaricides [105]. A rabbit serum containing polyclonal antibodies against GST from *R. microplus* reacted with the recombinant GST of *H. longicornis* and *R. appendiculatus*, suggesting that the tick GSTs could be a constituent of a universal vaccine that protects against more than one tick species [106]. Based on this preliminary study, Parizi and coworkers (2011) isolated GST from *H. longicornis* and produced recombinant GST and used this to vaccinate cattle against *R. microplus* [107]. This provided protection to cattle against *R. microplus* with an efficacy of 57%. The recombinant GST protein provided partial cross-protective immunity in the host, suggesting that the protein protective capacity of GST is not sufficient, and thus the use of this protein in a single-antigen vaccine would appear not to be effective in preventing tick infestation [107].

### 2.4. Malpighian-Associated Antigen Candidates 

In living organisms, 5′-nucleotidases are a widely distributed group of enzymes in various tick species. There are considerable similarities between the 5′-nucleotidase of ticks and the enzymes that are present in vertebrates, and a range of putative functions are carried out by these enzymes, including involvement in the purine salvage pathways [108]. Among ticks, this group of enzymes is found in many different tissues, such as the gut, salivary glands, and ovaries. However, they are most abundant in the Malpighian tubules, particularly on the surface of the Malpighian tubules and ovarian cells. The features of 5′-nucleotidases indicate that they are a potential target for antibodies [109]. Hope et al. investigated 5′-nucleotidases for their possible involvement in host immunization, and they found that injections of recombinant 5′-nucleotidase alone in sheep caused a significant upregulation in anti-nucleosidase antibodies, suggesting that they may be good antigen for the development of a vaccine [104]. However, when the same group of researchers analyzed their functions as antigens in cattle, there was no rise in antibody levels. Therefore, 5′-nucleotidases were not investigated further as antigens for vaccine development. However, a recent study conducted by another group of researchers has found that the level of 5′-nucleotidase/apyrase production increases in *O. erraticus* after feeding [110]. Furthermore, they suggested that blocking of the apyrase function by host immunization with a recombinant apyrase protein can strongly reduce feeding in *O. moubata* ticks, demonstrating that 5′-nucleotidases/apyrases are potentially promising candidate antigens for the development of an anti-tick vaccine [110,111].

### 2.5. Tick-Cement-Associated Antigen Candidates 

Tick cement is a mixture of glyco and lipoproteins secreted into the host via tick saliva shortly after attachment to the host, and is a valuable source of tick-derived antigens for vaccine development [112]. In addition to adhering tick mouthparts to the host skin [113], tick cement has been shown to act as a depot for *B. burgdorferi* sensu lato (s.l.) and the tick-borne encephalitis virus [114,115]. So far, various antigens have been identified and characterized from tick cement that have also been shown to be effective in controlling tick infestation and tick-borne diseases. 

Truncated constructs of 64P (64TRPs), a 15 kDa cement protein secreted by the salivary glands of *Rhipicephalus appendiculatus*, showed cross-protection against *Rhipicephalus sanguineus* and *Ixodes ricinus* by targeting antigens in the midgut and salivary glands, causing mortality in adults ticks and nymphs. The vaccination of tick-naïve hosts with recombinant 64P significantly reduced the number of nymphal and adult tick infestations, resulting in 48% nymphal and up to 70% adult mortality, with some effects on engorgement weight and egg masses as well [116]. From these results, it appears that this protein is a broad-spectrum vaccine antigen and is effective against adult and immature stages of different tick species, including *I. ricinus* [100]. This cement antigen performed a dual function (i) as a vaccine in hamster, guinea pig, and rabbit models by impairing attachment and feeding and (ii) by cross-reacting with the “concealed” midgut antigens, ultimately causing the death of engorged ticks [100,117]. This antigen not only boosts antibody titers in response to tick infestation, but also has cross-reactivity with different tick tissues; therefore, it combines the benefits of both “concealed” and “exposed” antigens [118].

**Table 1:** List of antigen candidates being studied at the pre-clinical or clinical stages for controlling ticks.

Antigen Candidate	Characterization	Feature of Antigen	Species	Experimental Animal	Efficacy or Reduction (%)	References
**Egg-associated antigens**						
BYC	Boophilus yolk pro-cathepsin	Native protein	*R. microplus*	Cattle	14–36	[24]
		Recombinant protein	*R. microplus*	Cattle	25.24	[26]
VTDCE	Vitellin-degrading cysteine endopeptidase	Native protein	*R. microplus*	Cattle	21	[31]
Vitelin	Vitelin	Native protein	*R. microplus*	Sheep	68	[28]
GP80	Glycoprotein	Native protein	*R. microplus*	Sheep	68	[28]
HH-GP80	Hexahis-GP80	Native protein	*R. microplus*	Cattle	72.80	[65]
FER1	Ferritin	Recombinant protein	*H. longicornis*	Rabbit	34	[72]
FER 2	-	Recombinant protein	*H. longicornis*	Rabbit	49	[72]
FER 2	-	Recombinant protein	*P. schulze*	Guinea pigs		[76]
FER 2	-	Recombinant protein	*I. ricinus*	Cattle	63–98	[75,119]
FER 2	-	Recombinant protein	*H. anatolicum*	Cattle	51.2–51.7	[74]
FER 2	-	Native protein	*R. microplus*	Cattle	64	[119]
BmTI N-terminal	Trypsin inhibitor	Synthetic peptide	*R. microplus*	Cattle	18.4	[64]
RmLTI		Recombinant protein	*R. microplus*	Cattle	32	[66]
**Gut associated antigens**						
Bm95	*B. microplus* 95, peptidase	Recombinant protein	*R. microplus*	Cattle	81.27–89	[34,120]
Bm4912	Glycoprotein	Synthetic peptide	*B. microplus*	Cattle	72.4	[121]
Bm7462^®^	Glycoprotein	Synthetic peptide		Control	81.05	[121]
Bm19733	-	Synthetic peptide	*R. microplus*	Cattle	35.87	[121]
Bm7462^®^	-	Recombinant protein		Cattle	72.4	[122]
Ba86	*B. annulatus* 86 protein	Recombinant protein	*R. microplus*	Cattle	71.5	[123]
Haa86	*H. anatolicum* 86 protein	Recombinant protein	*H. a. anatolicum*	Cattle	36.5	[124]
TROSPA	Tick receptor for outer surface protein A	Recombinant protein	*R. microplus*	Cattle	0	[14]
RmAQP1	Aquaporin 1	Recombinant protein	*R. microplus*	Cattle	68–75	[62]
RmAQP2	Aquaporin 2	Synthetic peptide	*R. microplus*	Cattle	25	[92]
OeAQP, OeAQP1	Aquaporins	Synthetic peptides	*O. erraticus*	Rabbit	4.6	[94]
GST	Glutathione S-transferases	Recombinant protein	*H. longicornis*	Cattle	57	[107]
ATAQ	Peptidase	Synthetic protein	*R. microplus*	Cattle	35	[125]
5′-nucleotidase		Recombinant protein	*R. microplus*	Cattle	0	[104]
**Salivary gland associated antigens**						
RmSUB	Subolesin, trancription factor	Recombinant protein	*R. microplus*	Cattle	51–75	[99,100]
BmSUB	Subolesin	Recombinant protein	*R. microplus*	Cattle	37.2–44.1	[101]
Bm91	*B. microplus* 91	Recombinant protein	*R. microplus*	Cattle	6	[35]
FSP	Flagelliform silk protein, Glycoprotein	Recombinant protein	*R. microplus*	Cattle	62	[14]
UBQ	Ubiquitin	Synthetic peptide	*R. microplus*	Cattle	55	[99]
**Cement-associated antigens**						
64TRPs	Truncated constructs of 64P		*R. appendiculatus*	Rabbits	40–70	[116]
**Multi-antigen**						
Rm39 + Rm76 + Rm180 + Rm239	*R. microplus* 39 + *R. microplus* 76 + *R. microplus* 180 + *R. microplus* 239	Recombinant salivary gland proteins	*R. microplus*	Cattle	73.2	[51]
SUB + Bm86	Subolesin and *B. microplus* 86 dual vaccine	Recombinant salivary gland (SUB) and gut (Bm86) protein	*R. microplus*	Cattle	97	[126]
SUB + IV	Subolesin + heat-inactivated *Mycobacterium bovis*	Recombinant salivary gland protein and inactivated mycobacterium	*R. microplus*	Cattle	65	[127]
Bm95-MSP1a	*B. microplus* 95-Major surface protein 1a	Recombinant protein	*R. microplus*	Control	64	[102]
RmLTI-BmCG-LTB	*R. microplus* larvae trypsin inhibitors, *B. microplus* Campo grande heat-labile enterotoxin B	Recombinant protein	*R. microplus*	Cattle	55.6	[128]
UBQ-MSP1a	Ubiquitin-major surface protein 1a	Recombinant protein	*R. microplus*	Cattle	0	[102]
SUB-MSP1a	Subolesin-Major surface protein 1a	Recombinant protein	*R. microplus*	Cattle	81	[102]
Q38		Recombinant protein	*R. microplus*	Cattle	75	[14,129]
EF1a-MSP1a	Elongation factor 1 alpha-Major surface protein 1a	Recombinant protein	*R. microplus*	Cattle	38	[102]
BrRm-MP4	Reprolysin *R. microplus*-Metaloprotease 4	Recombinant protein	*R. microplus*	Cattle	60	[50]
pcDNA3.1-HlLIP	Plasmid-Lipocalins	Recombinant plasmid	*H. longicornis*	Rabbit	30	[130]

## 3. The Types of Anti-Tick Vaccines 

Ticks are the most prevalent arthropod parasites that feed on humans and livestock and transmit diseases [19]. Zoonotic diseases account for more than 60% of all infectious diseases affecting humans, and it is estimated that 22.8% of these infections are transmitted by tick vectors [131]. There are also massive economic losses for livestock farmers worldwide due to the diseases vectored by ticks that affect their livestock [132,133]. 

**Figure 2:**
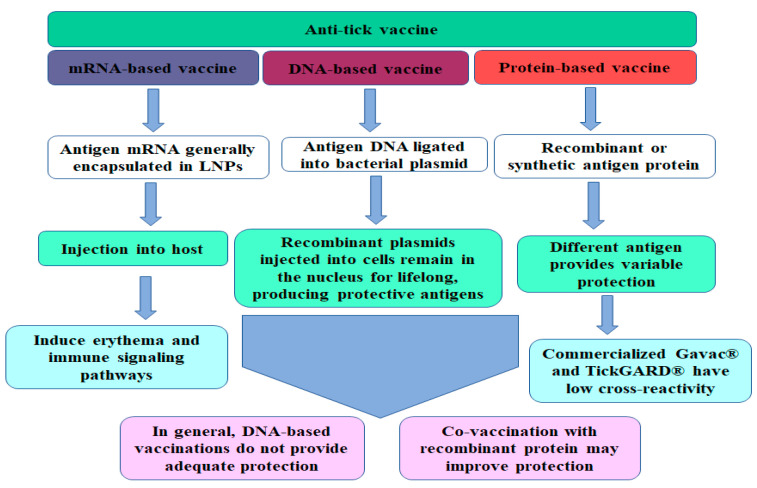
A schematic diagram representing the progress and development that has been made in the development of different anti-tick vaccines.

Therefore, it is relevant to control ticks to reduce the socio-economic burden. The control of ticks has become challenging, as ticks can develop resistance to commercially available acaricides [134,135,136]. Innovative environmentally sound control technologies are needed due to concerns about the safety of acaricides for workers, food, and the environment and the rising costs associated with acaricide discovery, development, and marketing. Vaccines have many advantages over acaricides since they are generally non-toxic, non-polluting, and less expensive compared to chemicals. However, they tend to be very species-specific in nature. The pharmaceutical industry can play a crucial role in supporting research to develop a vaccine that can provide maximum protection to the host and is effective against multiple tick species [133,137]. Vaccine research programs are underway in various countries and the remainder of this article will focus on the progress of new vaccine technologies and on those that are already available (Figure 2). As with chemical applications, vaccine resistance cannot be ignored, and existing vaccines can be modified using sequencing and cloning procedures to isolate new antigens or change existing antigens to restore their efficacy. It is also possible to include two or more unrelated antigens in a single vaccine product to reduce the risk of resistance developing to any single antigen or antigenic determinant. 

### 3.1. DNA-Based Anti-Tick Vaccines

The invention of the DNA vaccine and its use has raised safety concerns; in particular, the possibility of stable transfection of genetic material (DNA) into somatic or even germ cells, which may result in altered gene expression and mutations. An extrachromosomal plasmid-encoding luciferase vector was detectable in skeletal muscle for more than 19 months following intramuscular treatment [138]. Furthermore, intramuscular injection after electroporation greatly increased the overall transfection rate. The chromosomal integration of vector DNA at random sites is related to any increase in the transfection rate [139]. According to these studies, the integration frequency was well below the number of spontaneous gene mutations. However, Manam et al. found that the majority of plasmid DNA administered into the skeletal muscles of different rodents remained at the injection site. Minor fractions were also detected in the gonads, but were not integrated into the genome [140]. Repeated intramuscular application of a luciferase-encoding reporter vector in primates resulted in long-term reporter expression but induced no anti-DNA antibodies [141,142]. In spite of this, it should be noted that the aforementioned and additional safety concerns relating to DNA vaccines should be considered regarding their translation into clinical practice [143].

Over the last two decades, various groups of researchers have focused on developing a DNA vaccine to control tick infestation, and so far, several DNA vaccines have been introduced to immunize hosts [130,144,145]. DNA vaccines, which differ from traditional protein-based vaccines in that they are based on bacterial plasmids that encode antigenic proteins and the transcription is controlled by efficient eukaryotic promoters, have the advantages of a simple design, high stability, and safe administration [143]. In DNA vaccination, the injected plasmid DNA molecules are thought to actively enter the nucleus and remain there lifelong as episomal DNA, generating the protective antigens continuously for as long as the cell is alive [146]. The problem of repeated boosting to maintain a high antibody titer could be solved by the continuous synthesis, processing, and presentation of antigens to T cells in vivo in DNA-vaccinated animals. Furthermore, since a DNA vaccine only contains plasmid DNA and has no contaminating proteins, it seems plausible that receiving multiple or repeated vaccinations would not result in an immune reaction to the vector DNA [147]. The expressed antigen can be presented by MHC class I and II complexes, which can induce CD4+ and CD8+ T cells, stimulating cellular and humoral immune responses, respectively [148]. 

The evidence came from the study of De Rose et al. [144], in which Merino crossbred sheep were immunized against *B. microplus* using a DNA vaccine. When a plasmid containing full-length gene sequence of Bm86 was administered either alone or with a plasmid carrying the ovine genes for the cytokines, granulocyte macrophage colony-stimulating factor (GM-CSF) or interleukin (IL)-1beta induced a relatively low level of protection against subsequent tick infestation. In addition, co-vaccination with Bm86 and GM-CSF plasmids resulted in a statistically significant reduction in the fertility of ticks. In all groups injected with the Bm86 DNA vaccine, antibody titers against Bm86 were low. In addition, a low level of antigen-specific stimulation of peripheral blood lymphocytes occurred in these groups. DNA vaccination, however, resulted in a strong subsequent antibody response following a single injection of recombinant Bm86 protein in adjuvant. The production of antibodies, however, did appear to be slightly less effective than following two vaccinations with recombinant proteins. 

Furthermore, many other researchers investigated the efficacy of DNA-based antigens to immunize hosts against tick infestations. For example, Sayed et al. [149] extracted DNA from *Argas persicus* eggs and used it to immunize chickens with doses of 200–800 μg DNA/kg chicken body weight. The outcome was supportive, as the feeding success of ticks reduced by 74.64% (50 µg DNA/kg chick body weight) and 89.39% (100 µg DNA/kg chick body weight) when they were exposed to DNA-immunized chicken. In addition, the authors reported that the serum of chickens immunized with DNA has activity against the gut proteins of *A. pericus*; however, further analysis using other tick species indicated that the serum activity is species specific. The electrophoretic pattern of the immunized chicken serum showed three new protein bands, which were assumed to be involved in the development of the immune defense of the chicken against ticks [149]. Afterwards, many studies focused on antigen-specific DNA vaccination; however, their protection level varied with different types of antigens. It has been shown that BALB/c mice injected with Plasmid pBMC2-encoding antigen Bm86 showed resistance against *Boophilus microplus*. A higher dose of vaccination induced Anti-Bm86 antibody production and higher interleukin (IL) levels (IL-4, IL-5, and IL-12 (p40)) and interferon-gamma (IFN-γ) levels in the sera of mice immunized with pBMC2. Mice immunized with pBMC2 showed antigen-specific stimulation of splenocytes according to the incorporation of bromodeoxyuridine and IFN g secretion. Application of this vaccine in livestock caused antibody production, suggesting that Bm86 DNA vaccination induces a strong immune response against *B. microplus* [150]. Another study evaluated the immune protection elicited by recombinant plasmids encoding Paramyosin (Pmy) of *H. longicornis* (pcDNA3.1(+)-Pmy) in rabbits. The rabbits developed a high level of IgG, suggesting that a humoral immune response is induced by vaccination. Some ticks (27.31%) that fed on the vaccinated rabbits died, whereas the remaining ticks’ average engorgement weight and the oviposition of female adults were reduced by 36 and 39%, respectively. Thus, it seems that a Pmy DNA vaccine can induce an effective humoral immune response however it is provided and partially protect rabbits against *H. longicornis* infection [151]. Interestingly, a multi-epitope DNA vaccine incorporating both CD4+ and CD8+ cytotoxic T lymphocyte epitopes provided 100% protection to sheep under laboratory conditions against *Ehrlichia ruminantium.* However, the results were not repeated under field conditions. In this study, pLamp co-administration with MPL via the intramuscular route, in addition to topical application, provided protection to the sheep of up to 60% against ticks by inducing activation of memory T cell responses [152].

A number of other antigens, such as Salp14 and lipocalins, either alone or in combination with other antigens have recently been evaluated as DNA vaccines, which further provides hope of developing a DNA vaccine against ticks [130,153]. It has been shown that the salp14 DNA vaccine elicited erythema at the tick bite site after the tick challenge [153]. Similarly, a lipocalins (LIP) vaccine comprising the recombinant plasmid pcDNA3.1-HlLIP of the LIP homologue from *H. longicornis* (HlLIP) was applied to immunize a rabbit host. Although this application induced humoral immunity of the host and also influenced the engorgement weight, oviposition, and hatchability of *H. longicornis*, the efficacy was too low, suggesting that this antigen is not suitable for vaccines as it provides partial protection to the host [130].

Finally, the main objective of developing a DNA vaccine should be to design the vaccine in such a way that it polarizes the immune response of the host towards the Th2 response, since the humoral immune response plays a major role in tick immunity. In cattle that had been vaccinated with *B. microplus* midgut antigens, the levels of specific IgG1, which are modulated by Th2 cells, were found to correlate with the protection. There is a possibility that if the secretory signal sequence is placed appropriately downstream of the target gene, then the target antigen could be secreted out to the extracellular compartment and induce a greater humoral immune response. It is also likely that the selection of Th2 cells would be favored if they are coinfected with other immunomodulatory genes such as IL4 and IL10.

### 3.2. mRNA Vaccine 

In recent years, many attempts have been made to discover new protective antigens that can be used in the development of an anti-tick vaccine, with numerous improvements. To test the efficacy of the vaccine candidate, recombinant proteins, regardless of whether or not they are associated with other proteins or adjuvants, have been the platform of choice for testing their efficacy through the use of model organisms. Due to the ease with which DNA and mRNA vaccine platforms can be generated, there has been significant developments in the use of genetic (DNA and mRNA) vaccine platforms in recent years [130,145]. An mRNA vaccine encoding a cocktail of tick salivary proteins induced “tick immunity” in guinea pigs, thereby remarkably reducing the transmission of tick-borne *Borrelia burgdorferi*, the causative agent of Lyme disease (borreliosis). Despite the fact that many tick antigen candidates have been demonstrated to elicit immune responses in a host, it has not yet been possible to replicate robust tick immunity using vaccination. There is a possibility that this may be due to the fact that at different stages of feeding, the composition of salivary proteins in ticks may alter dynamically, possibly to manage host internal changes. This information was provided in a recent study, in which a group of researchers identified and rationally selected 19 salivary proteins of the black-legged tick *I. scapularis*, which is a common vector for Lyme disease in humans. They engineered nucleoside-modified mRNAs that encode for these proteins. In order to produce the mRNA–LNP vaccine 19ISP (19 ixodes salivary proteins), these were encapsulated in equal amounts in lipid nanoparticles (LNPs). To evaluate the impact of the vaccine on the feeding behavior of *I. scapularis*, guinea pigs were immunized intradermally three times in 4-week intervals, which resulted in robust antibody responses to at least ten of the encoded antigens. In the next step of the experiment, the animals were challenged with uninfected *I. scapularis* nymphs. The animals that had been vaccinated developed considerable erythema within 24 h. Furthermore, ticks on animals that had been vaccinated fed poorly and began to detach by 48 h, with 80% of ticks detached from vaccinated animals after 96 h, compared with 20% on animals that had not been vaccinated. To further examine whether the altered feeding behavior affects the transmission of pathogens, *B. burgdorferi*-infected *I. scapularis* nymphs were placed on guinea pigs that were vaccinated either with 19ISP or with an mRNA vaccine encoding firefly luciferase. Each of these animals received three ticks that were infected. Considering that humans are likely to remove a tick that causes erythema-related itching, the ticks were detached in a double-blind manner as soon as redness appeared. A total of 46% of the control animals were infected with *B. burgdorferi* three weeks after the challenge, whereas none of the vaccinated animals were infected with this pathogen. A gene expression analysis has shown that the vaccine activated several immune pathways, including T and B cell receptor, chemokine, FcεRI, and IL-17 signaling, as well as natural killer cell-mediated toxicity. Moreover, bite site analyses also showed that the vaccine had induced T cell responses [154]. 

Concurrently, the same group of researchers used Salp14 as a model antigen to examine tick immunity using mRNA lipid nanoparticles (LNPs), plasmid DNA, or recombinant protein platforms [153]. In this study, vaccination including the nucleoside-modified mRNA lipid nanoparticles encoding (mRNA-LNPs) Salp14 was delivered intradermally, with two boosts every 4 weeks. The development of Salp14-specific antibodies was compared among the different immunization strategies. Salp14 mRNA immunization was the platform that induced the strongest humoral response compared to DNA and protein vaccination. Guinea pigs immunized with the salp14 mRNA elicited the most robust, and intense erythema was observed at the bite site in all the immunization groups; however, it did not affect the rate of tick detachment and did not alter engorgement weights [153]. A tick vaccine should induce erythema to be effective, and one approach to change later aspects of tick feeding, including attachment and engorgement, is to use a vaccine that contains several salivary tick antigens [154]. Therefore, it seems that immunization with nucleoside-modified mRNA-LNP salp14, which can be used as a potential vaccine candidate, can lead to higher antibody titers and an earlier and higher degree of redness than immunization with either DNA or protein, which suggests that Salp14 could be a good candidate for a vaccine, either alone with optimizations or in combination with other candidate antigens [154]. 

On the whole, it appears that a multivalent mRNA vaccine may have the ability to elicit tick resistance in laboratory animals such as guinea pigs and to prevent tick infestation and tick-borne infection, probably by limiting the time duration of tick feeding on their host. It has also been suggested that a mRNA–LNP formulation which enables slow, continuous antigen delivery, may mimic natural tick bites. If this strategy can be translated to humans, it would be the first vaccine that does not directly target a pathogen or microbial target, but instead its vector. Moreover, since anti-tick vaccines are still being developed to assist humans in the prevention of the transmission of tick-borne diseases, the strategy of immunization and the selection of antigens for immunization need to be taken into consideration.

### 3.3. Protein-Based Vaccines 

Some protein-based vaccines are commercially available and have been shown to be effective. A vaccine program was first started in the 1970s when researchers began to experiment with two different types of vaccine formulations in order to immunize the host against the tick (*D. andersoni*) at the time. The first included antigens obtained from the gut and ovary, while the second included all the internal organs extracted from semi-engorged *D. andersoni* females. This study discovered that antibody-mediated immune responses are activated against tick intestinal tissue when the cattle host is inoculated with extracts obtained from adult *R. microplus* females [155]. This initial study to evaluate the effectiveness of vaccine formulation encouraged researchers around the globe to focus on the development of a vaccine for the control of ticks and tick-borne diseases. Thus, in the 1980s, two separate groups of researchers carried out the first scientific investigations using vaccine formulations to analyze the immune response of bovines against *R. microplus* [156]. 

Following the above studies, it was found that a tick gut-associated glycoprotein can induce immunoprotection in the host [157]. In a subsequent study, the same research group isolated a glycoprotein with a molecular weight of 89 kDa, which was named Bm86 and reported to be associated with the gut cells of *R. microplus* [158,159]. The Bm86 recombinant protein was produced on a large scale using a yeast expression system. So far, only the Bm86-based vaccine is commercialized with different brand names, for example, it is sold in Australia with the TickGARD^®^ brand name and in Cuba under Gavac^®^ [160,161]. These vaccines are largely used in different countries to reduce the tick pressure on cattle. It has been shown that the use of these vaccines can reduce the tick population by up to 74% and their overall efficacy ranges from 51% to 91%, which varies with the tick population and nutritional condition of the cattle [160,161,162,163]. There is evidence that some Columbian, Mexican, and Brazilian *R. microplus* tick strains exhibit lower overall efficacy compared to Cuban and Australian *R. microplus* tick strains, and even the Argentinian *R. microplus* strain A seems to be resistant to vaccination with Bm86 [34,164]. Further analysis was conducted on the variation in the efficacy of the Bm86 vaccine on populations of the same tick species in different parts of the world, and it was concluded that different populations of ticks most likely have a polymorphism in Bm86 antigen genes in terms of the amino acid sequence of the gene, and this is the main reason that existing Bm86-based vaccines are not so efficacious. For example, there was a polymorphism in the gene homologous to Bm86 (designated as Bm95) identified in tick populations in Argentina, resulting in differences in the sequence of the Bm86 between tick populations such as those found in Cuba and Australia, which may explain why the Bm86 vaccine was not as effective against the Argentine tick [34]. Considering the resistance problems of the Bm86 vaccine, researchers produced a recombinant Bm95 vaccine that has proven to be highly effective, with an overall efficacy of 89% in Cuba and Argentina and 81% in India in terms of reducing tick infestation [34,102,120,165].

Besides the above-mentioned commercial vaccine and its efficacy trial, many other studies have focused on further improving the efficacy of the Bm86-based vaccine. Some recent studies have synthesized peptides, including SBm4912, SBm7462^®^, and SBm19733, which were obtained from Bm86, and also produced an rSBm7462^®^ recombinant peptide, and analyzed their efficacy. The percentage efficacy of these peptides ranged from 35.87% to 81.05%, suggesting that these peptides, in particular, SBm7462^®^ and rSBm7462^®^, play a crucial role in inducing host immunity and can be commercialized as they are highly effective in terms of reducing tick infestation [121,122]. Furthermore, in an effort to improve the Bm86 recombinant protein vaccine effectiveness, recently, Lapisa S.A. has introduced a Bm86-based Bovimune Ixovac^®^ vaccine in Mexico. However, this vaccine has not been studied in terms of its effectiveness against ticks; therefore, studies are needed to determine its effects on different tick populations to determine its efficacy. 

A Bm86 homologue-based vaccine (TickGard) appears to be suitable, as this vaccine has a broad application and can trigger cross-reactive antibodies in different tick species, such as *Rhipicephalus sanguineus*, *Hyalomma anatolicum anatolicum*, *Rhipicephalus* (Boophilus) *decoloratus*, *Rhipicephalus* (Boophilus) *annulatus*, and *Hyalomma dromedarii* [166,167,168]. However, this vaccine has been shown to be unable to induce cross-reactive protection in some other tick species (e.g., *Rhipicephalus appendiculatus*, *Amblyomma variegatum*, and *Amblyomma cajennense*) [169,170]. It is interesting to note that the Bm86 vaccine has a 100% efficacy against *R. annulatus*, resulting in greater efficacy than the reported efficacy of the homologous vaccine with *R. microplus*. The reason for this might be due to physiological factors (e.g., less blood engorgement and lower levels of protease activity in the body) or tick genetic factors. It is possible that these factors influence BM86 protein levels or tick physiological processes such as feeding and protein degradation, leading to more efficient antibody–antigen interactions [171]. Bm86 vaccination provides excellent protection against *R. microplus* ticks, but it is challenging to extrapolate these experiences to an Ixodes tick vaccine. In contrast to *I. ricinus* and *I. scapularis*, *R. microplus* is a single host tick that feeds exclusively on cattle [168]. It also has a brief life cycle, does not molt and finds a new host when the blood meal is finished. The efficacy of the Bm86 vaccine was investigated in cows that had been exposed to *R. microplus* tick larvae, and measurements were made of the parameters relating to tick immunity on the engorged adult females that dropped off following vaccination. Thus, the measured protection is the sum of the influence on two molting periods and three tick stages. It has been shown for *R. microplus* that Bm86 vaccination causes damage and subsequently reduces the engorgement weight in adult female ticks [172]; nevertheless, the relative influence of Bm86 vaccination on the immature life stages of *R. microplus* is not precisely known. Bm86 homologues have also been isolated and identified from Ixodes ticks. *I. ricinus* contains two homologues of Bm86, Ir86-1, and Ir-86-2, and *I. scapularis* also has two homologues of Is86-1 and Is86-2 [173]. A subsequent study explored that vaccination of recombinant Ir86 proteins although enhanced the serum IgG titers against recombinant Ir86 proteins; however, the antibodies were not able to protect rabbits against *I. ricinus* challenge; neither the number of attached ticks nor tick weights were reduced [174]. Therefore, vaccination against Bm86 homologues in Ixodes is not considered to be an effective approach to control Ixodes ricinus populations, despite the fact that Bm86 vaccination has a clear effect against *R. microplus*. Even though the Bm86 vaccine has shown considerable success, it is critical to understand that the vaccine is unlikely to replace acaricides because it lacks the “knock-out effect” associated with acaricides. In spite of this, field experience has shown that the use of Bm86 considerably reduces the requirement of applying acaricide treatments in the field. For example, Cuba’s tight regulation of its tick control program led to a reduction in the amount of acaricide used in the country by 87%, which is comparable to the results of a recent study conducted in Venezuela [161,175,176]. Furthermore, the use of the Bm86 vaccine has also considerably decreased tickborne diseases, including Bovine anaplasmosis and Bovine babesiosis. The application of this vaccine has also enhanced the productivity of livestock, e.g., cattle, and consequently reduced the economic losses of farmers [160]. The above aspects of Bm86 vaccination show that it is a highly cost-effective method compared to chemical applications in dealing with tick infestations. As such, this vaccine may prove to be highly useful in reducing tick-borne diseases as well as in improving the management of tick outbreaks on livestock farms to decrease tick-borne diseases.

## 4. Concluding Remarks

Ticks feed on blood for development, growth, and reproduction, and they are responsible for the transmission of various tick-borne diseases. *Ixodes* ticks, for example, transmit a large number of pathogens, including bacteria, protozoa, and viruses. It is important to note that anti-tick vaccines reduce tick infestation and prevent pathogen transmission, and in addition are safer than chemical control, which may have negative side effects. A number of novel antigens from a variety of tissues/organs have been identified and their efficacy has been examined in laboratory animals, which has led to important progress towards developing a vaccine against ticks with no reported side effects. Vaccines based on the Bm86 protein have been commercialized in various countries, and their use against *R. microplus* has shown that vaccination against ticks can be efficiently used. However, Bm86-based vaccines are not equally effective against other tick species, such as *Ixodes* ticks. It has been shown that vaccination with the Bm86 homologue of *I. ricinus* does not have any effect on tick feeding [174]. Additionally, because *R. microplus* only feeds on one host, larvae were used to challenge cows, which resulted in fully engorged adult female ticks, which not only reduced the number of ticks but also had a significant impact on all three life stages of the tick. In contrast to *R. microplus*, *Ixodes* ticks change hosts throughout their life cycle, and therefore *Ixodes* tick vaccination requires the effective prevention of attachment and/or feeding of ticks during one blood meal on one host. Nonetheless, vaccination against *Ixodes* ticks seems possible and realistic. It is thus important to note that cross-protection of the host can be a challenging task with this type of vaccine. There are some other proteins, besides Bm86, that might also have therapeutic potential as immunosuppressive or anticoagulant agents [39,61]. Recent advances in genomics and proteomics have enabled us to discover novel antigens and employ molecular techniques to manipulate identified proteins and test new vaccines considerably more quickly and cost-effectively than in the past. DNA vaccination is also an excellent option; however, in general, this type of vaccination can only lead to low levels of antigen expression and limits the non-professional antigen-presenting cell activating CD4+ T helper cells via the MHC class II pathway [177]. However, DNA vaccination not only provides significant protection to the host but is also considered to provide cross-protection against ticks if it is followed by a chimeric vaccine or recombinant protein vaccination [178]. Furthermore, mRNA-LNPs may help in the elicitation of erythema at the tick bite site, which is one of the most important early indicators of acquired tick resistance. mRNA-LNPs containing tick genes are a useful platform for the development of vaccines that can potentially prevent selected tick-borne diseases [153,154]. Both DNA and mRNA vaccination also seem to be effective strategies and there is future hope that a mRNA or DNA vaccination for the control of tick infestation and tick-borne diseases may be developed and also provide cross-protection. However, so far no vaccine has been commercialized, indicating that further studies are needed to determine the efficacy of increasingly more antigens for their use to develop a DNA or mRNA vaccine. The identification and characterization of novel tick vaccine candidates can prevent tick feeding and pathogen transmission. Using these antigens in vaccines for domesticated animals and wildlife, let alone humans, remain a challenge. 

## Data Availability

Not applicable.
